# Functional characterization and evolution of olfactory responses in coeloconic sensilla of the global fruit pest *Drosophila suzukii*

**DOI:** 10.1186/s12915-025-02151-9

**Published:** 2025-02-21

**Authors:** Qi Xue, Kazi Sifat Hasan, Omar Dweck, Shimaa A. M. Ebrahim, Hany K. M. Dweck

**Affiliations:** 1 Department of Entomology, The Connecticut Agricultural Experiment Station, New Haven, CT 06511 USA; 2 Department of Molecular, Cellular and Developmental Biology, Yale University, New Haven, CT 06511 USA; 3 Wilbur Cross High School, 181 Mitchell Dr, New Haven, CT 06511 USA

**Keywords:** *D. suzukii*, *D. melanogaster*, Olfaction, Coeloconic sensilla, Orco, Phenylacetaldehyde, Attractant

## Abstract

**Background:**

When a species changes its host preference, it often requires modifications in its sensory systems. Many of these changes remain largely uninvestigated in the global fruit pest *Drosophila suzukii* (also known as spotted wing Drosophila, SWD). This species, which shares a last common ancestor with the model organism *D. melanogaster*—a species that prefers overripe fruits— ~ 15 million years ago, has shifted its preference from overripe to ripe, soft-skinned fruits, causing significant damage to fruit industries worldwide.

**Results:**

Here, we functionally characterized the coeloconic sensilla in *D. suzukii* and compared their responses to those of its close relatives, *D. biarmipes* and *D. melanogaster*. We find that *D. suzukii*’s responses are grouped into four functional types. These responses are consistent across sexes and reproductive status. The odorant receptor co-receptor Orco is required for certain responses. Comparative analysis across these species revealed evolutionary changes in physiological and behavioral responses to specific odorants, such as acetic acid, a key indicator of microbial fermentation, and phenylacetaldehyde, an aromatic compound found in a diverse range of fruits. Phenylacetaldehyde produced lower electrophysiological responses in *D. suzukii* compared to *D. melanogaster* and elicited strong attraction in *D. suzukii* but not in any of the other tested species.

**Conclusions:**

The olfactory changes identified in this study likely play a significant role in the novel behavior of *D. suzukii*. This work also identifies phenylacetaldehyde as a potent attractant for *D. suzukii*, which can be used to develop targeted management strategies to mitigate the serious impact of this pest.

## Background

Adaptation to new environments often requires alterations in the nervous system, which can occur at both peripheral and central levels [1–13]. Elucidating these adaptive changes is crucial not only for understanding how animals alter their behaviors but also for gaining valuable insights that may facilitate the development of innovative strategies for monitoring and managing these animals.

The global fruit pest *Drosophila suzukii*, commonly known as spotted wing *Drosophila*, offers an excellent opportunity to investigate such changes, particularly those associated with its unique preference for ripe fruits. In contrast to *D. suzukii*, which poses a significant threat to fresh, soft-skinned fruits, its ancestors, including the model organism *D. melanogaster*, prefer fermented fruits, which hold no commercial value [4, 6, 11]. Olfaction, along with other senses, is thought to contribute significantly to this shift [4, 6, 11, 12, 14, 15].

In insects, there are two peripheral olfactory systems: one expressing odorant receptors (Ors) in combination with the obligatory odorant receptor co-receptor (Orco), and the other expressing ionotropic receptors (Irs) [16–18]. Prior research on *D. suzukii* has predominantly investigated adaptive changes in the Or-expressing olfactory system [14, 15, 19], while the Ir-expressing olfactory system has been less explored.

In the model organism *D. melanogaster*, the Ir-expressing olfactory system comprises four functional types of antennal coeloconic sensilla (ac1-ac4), in addition to some neurons in the sacculus, an invagination in the posterior region of the antenna [20–22]. The ac1, ac2, and ac4 sensilla house three neurons each, whereas the ac3 sensilla houses two neurons [20, 21]. All neurons within the coeloconic sensilla express members of the Ir gene family, and all neurons except ac3B respond to acids and amines [20, 21]. The ac3B neurons, in addition to expressing Ir8a, Ir25a, Ir76b, and probably an unknown Ir gene, also express Or35a in conjunction with Orco and respond to alcohols, aldehydes, ketones, and many other odorants [20–24]. Responses to nearly all odorants detected by this neuron depend on Or35a [22].

The antenna of *D. suzukii* also harbors coeloconic sensilla [25], yet the exact number of their distinct functional types remains unidentified*,* and whether responses to certain odorants in these types depend on Orco is unknown. Additionally, it is unclear if there are differences in odorant response profiles of these types between males and females or between virgin and mated females. Furthermore, it remains undefined whether *D. suzukii* and its close relatives differ in their electrophysiological and behavioral responses to the ligands of these types. In this study, we addressed these questions using the extracellular single-sensillum recording (SSR) technique, a *D. suzukii* Orco mutant line, and behavioral bioassays.

## Results

### Four functional types of coeloconic sensilla in *D. suzukii*

**Fig. 1:**
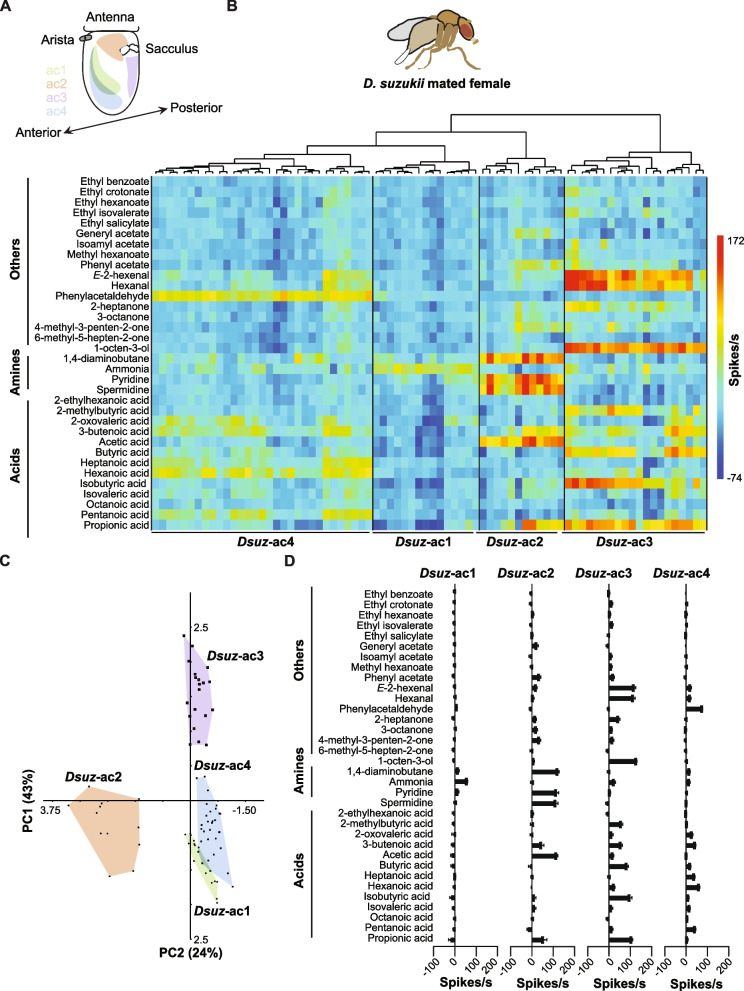
Functional types of coeloconic sensilla in *D. suzukii*. **A** Distribution of functional types of antennal coeloconic sensilla mapped manually after electrophysiological recordings. **B** Heatmap based on hierarchical cluster analysis of responses of coeloconic sensilla in mated females to a panel of 34 odorants. Each vertical row represents one coeloconic sensillum, and each horizontal column represents one odorant. Classification was carried out using Ward’s method. Odorants were diluted 10^−2^ in double-distilled water. Responses to the diluent control, water, were subtracted from each value. **C** Principal components analysis of responses of coeloconic sensilla to a battery of 34 odorants. PC1 and PC2 explain 43% and 24% of the variance, respectively. **D** Response profiles of functional types of coeloconic sensilla in mated females. Error bars represent means ± SEM. Sample sizes (*n*) range from 12 to 31, as shown in panel **A**. Responses to the diluent control, water, were subtracted from each value

To analyze the odorant response profiles of coeloconic sensilla in *D. suzukii*, functionally classify them, and map their location on the antenna, we examined a total of 78 different coeloconic sensilla in ~ 20 mated females (Fig. 1). These sensilla cover a significant portion of the available antennal surface (Fig. 1A). For this analysis, we used a battery of 34 odorants, resulting in a total of 2652 recordings (Fig. 1B). This battery included 13 acids, 4 amines, and 17 other compounds. Many of these odorants have been previously used to characterize coeloconic sensilla in other *Drosophila* species [9, 21, 22, 26, 27], and many, such as acids and polyamines (1,4-diaminobutane (also known as putrescine) and spermidine), have been identified in ripe fruits [28, 29].

We then quantified the responses by calculating the total number of spikes per sensillum, as reliable spike sorting to identify individual neurons was not feasible. This challenge is also noted in the coeloconic sensilla of other *Drosophila* species [8, 9, 21, 22, 27]. We then used hierarchical cluster analysis (Ward’s method) and principal component analysis (PCA) to classify the 78 coeloconic sensilla based on their responses to our battery of 34 odorants.

We found that the responses of the 78 coeloconic sensilla of *D. suzukii* mated females were segregated into four functional types (clusters) (Fig. 1B-D). These types are designated as ac1-ac4, akin to the nomenclature used in *D. melanogaster* and other *Drosophila* species [8, 9, 16, 21, 22, 27]. Each of these four functional types exhibits a distinct response profile (Bonferroni-corrected *p* ≤ 0.0024, *R* = 0.49; one-way ANOSIM based on Bray–Curtis similarity) (Fig. 1C).

The ac1 sensillum type is found in a region on the anterior antennal surface just ventral to the arista (Fig. 1A). This type is represented by 15 sensilla in our analysis. These sensilla gave strong excitatory responses exclusively to ammonia, with an average spikes/s of 57 ± 3 (Fig. 1B, D).

The ac2 sensillum type is situated around the sacculus and is represented by 12 sensilla in our dataset (Fig. 1A, B). These sensilla exhibited responses of more than 100 spikes/s to each of 1,4-diaminobutane (120 ± 8 spikes/s), acetic acid (115 ± 7 spikes/s), pyridine (114 ± 11 spikes/s), and spermidine (111 ± 12 spikes/s) (Fig.1B, D).

The ac3 sensillum type, represented by 20 sensilla in our screen, is located on the posterior side of the antennae (Fig. 1A, B). This type is the only coeloconic sensillum type in *D. suzukii* that showed excitatory responses to 1-octen-3-ol (128 ± 3 spikes/s), *E*-2-hexenal (117 ± 8 spikes/s), hexanal (114 ± 9 spikes/s), and 2-heptanone (44 ± 7 spikes/s) (Fig. 1B, D). These odorants typically activate neurons that express Ors in combination with Orco [20–22, 30, 31]. Additionally, these sensilla exhibited excitatory responses to several acids, including propionic acid (106 ± 6 spikes/s), isobutyric acid (97 ± 10 spikes/s), butyric acid (83 ± 8 spikes/s), 2-methylbutyric acid (57 ± 8 spikes/s), and 3-butenoic acid (55 ± 8 spikes/s) (Fig. 1B, D).

The ac4 sensillum type is located on the anterior side of the antenna, overlapping with ac1 in some regions (Fig. 1A). Our analysis included 31 sensilla of this type, all of which showed responses of 76 ± 3 spikes/s to phenylacetaldehyde, 61 ± 4 spikes/s to hexanoic acid, 43 ± 3 spikes/s to 3-butenoic acid, 42 ± 4 spikes/s to pentanoic acid, and 38 ± 4 spikes/s to heptanoic acid (Fig. 1B, D).

We also found that several compounds decrease the spontaneous firing activity of neurons within these coeloconic sensilla (Fig. 1B, D). For example, propionic acid decreased the spontaneous firing activity of neurons in ac1 sensilla by 20 ± 9 spikes/s, while pentanoic acid reduced the spontaneous firing activity of neurons in ac2 sensilla by 18 ± 5 spikes/s.

### Conservation of electrophysiological responses of *D. suzukii* coeloconic sensilla across sexes and reproductive status

We also conducted a parallel analysis focusing on *D. suzukii* males to investigate whether females and males exhibit divergent responses to certain odorants, particularly polyamines. Transcriptomic profiling of the chemoreceptor repertoire in both male and female *D. suzukii* revealed that *Dsuz*Ir76a exhibits female-biased expression [32]. In *D. melanogaster*, Ir76a serves as an olfactory receptor for detecting polyamines [21, 33].

**Fig. 2:**
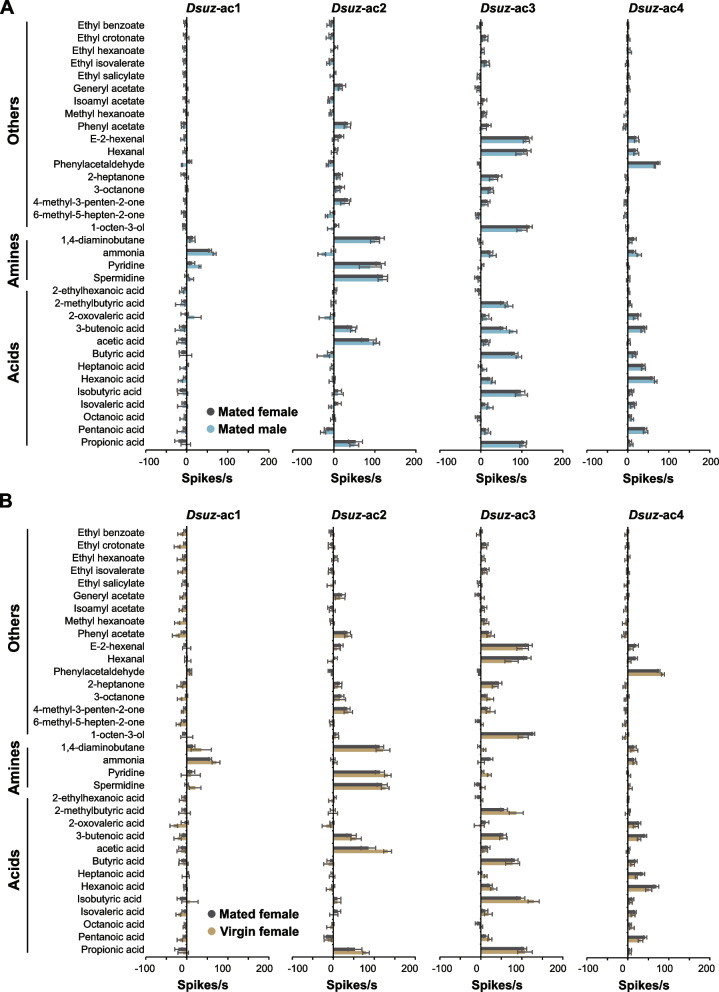
Responses of *D. suzukii* coeloconic sensilla across sexes and reproductive status. **A** Response profiles of functional types of coeloconic sensilla in mated females and males. Error bars represent means ± SEM. For mated females, sample sizes (*n*) range from 12 to 31. For males, sample sizes (*n*) range from 5 to 14. Responses to the diluent control, water, were subtracted from each value. **B** Response profiles of functional types of coeloconic sensilla in mated and virgin females. Error bars represent means ± SEM. For mated females, sample sizes (*n*) range from 12 to 31. For virgin females, sample sizes (*n*) range from 5 to 8. Responses to the diluent control, water, were subtracted from each value

We found that the distribution and odorant response profiles of the four functional types of the coeloconic sensilla were identical between the sexes (Fig. 2A). We observed no significant differences in the response to any of the tested odorants, including the two polyamines (1,4-diaminobutane and spermidine), between males and females (*p* > 0.05 for each odorant; Mann–Whitney test).

Although we did not observe differences in electrophysiological responses to polyamines between sexes, the female-biased expression of Ir76a may correspond to a difference in the number of neurons that express this receptor.

Next, we investigated whether mating influences the responses of coeloconic sensilla. Previous reports have shown that mating alters *D. suzukii* preferences, as mated females tend to prefer fruit volatiles, while virgin females favor fermentation volatiles [34, 35]. This observation could in principle be attributed in part to changes in peripheral physiology. Consequently, we compared the odorant response profiles of virgin and mated females and observed no differences (*p* > 0.05 for each odorant; Mann–Whitney test) (Fig. 2B). This finding is consistent with the conservation of the expression levels of antennal Ir genes between *D. suzukii* virgin and mated females [36].

Collectively, these findings suggest that the odorant response profiles of coeloconic sensilla in *D. suzukii* are conserved not only between males and females but also between virgin and mated females.

### Responses of ac3 sensilla to certain odorants require the *odorant receptor coreceptor Orco* in *D. suzukii*

Having shown that *D. suzukii* ac3 responds to odorants (1-octen-3-ol, 2-heptanone, hexanal, and *E*-2-hexenal) that typically activate Ors + Orco-expressing neurons, we sought to determine whether the responses to these odorants require Orco. Previous studies in *D. melanogaster* have indicated that these odorants activate ac3B neurons, which express Or35a along with Orco, and that this activation is dependent on Or35a [20, 22]. However, the role of Orco in this activation has not been extensively explored in *D. melanogaster*, *D. suzukii*, or any other *Drosophila* species. Only one study in *D. melanogaster* has reported that the responses to one concentration of hexanol in ac3B sensilla depend on Orco [23].

**Fig. 3:**
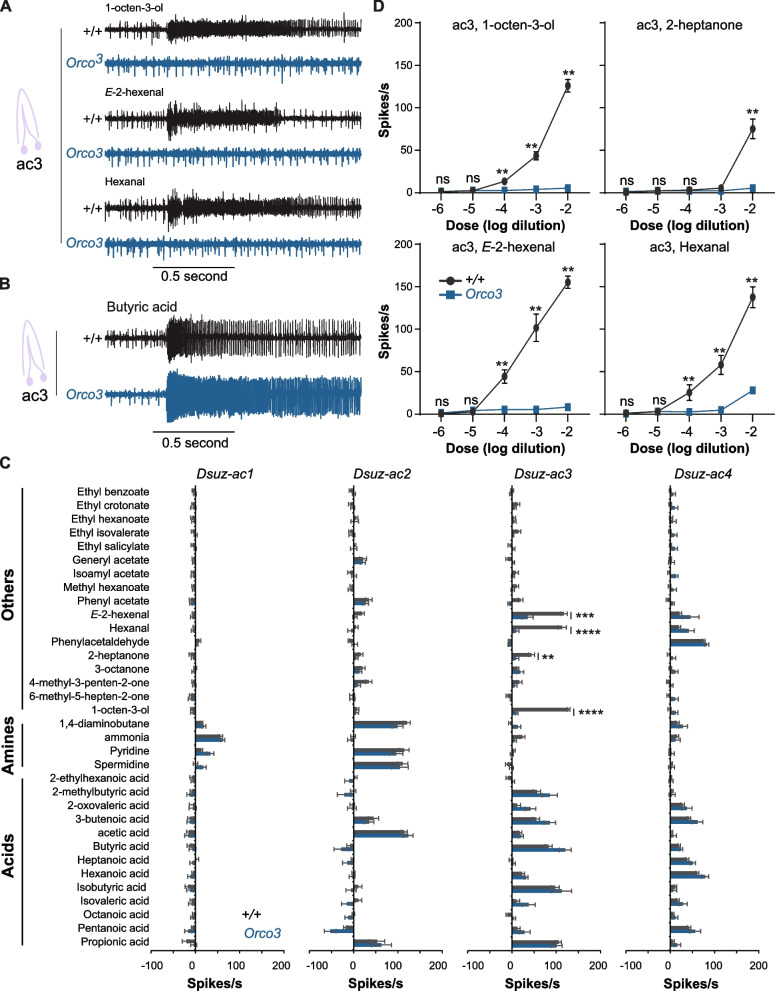
Responses of coeloconic sensilla in *D. suzukii* wild-type and *Orco*^*3*^ mutant mated females. **A** Example traces of electrophysiological responses of *D. suzukii* wild-type (+ / +) and *Orco*^*3*^ mutant mated females to 1-octen-3-ol (10^−2^ dilution), *E*-2-hexenal (10^−2^ dilution), and hexanal (10^−2^ dilution). **B** Example traces of electrophysiological responses of *D. suzukii* wild-type (+ / +) and *Orco*^*3*^ mutant mated females to butyric acid (10^−2^ dilution). **C** Response profiles of functional types of coeloconic sensilla in *D. suzukii* wild-type (+ / +) and *Orco*^*3*^ mutant mated females. Error bars represent means ± SEM. For *D. suzukii* mated females, sample sizes (*n*) range from 12 to 31. For *Orco*^*3*^ mutant mated females, sample sizes (*n*) range from 6 to 13. Responses to the diluent control, water, were subtracted from each value. Mann–Whitney test, **p ≤ 0.01, ****p ≤ 0.0001. **D** Responses of *D. suzukii* wild-type (+ / +) and *Orco*^*3*^ mutant mated females to different doses of each of 1-octen-3-ol, 2-heptanone, *E*-2-hexenal, and hexanal. Error bars represent means ± SEM. Responses to the diluent control, water, were subtracted from each value. Mann–Whitney test, **p ≤ 0.01, ****p ≤ 0.0001, *n* = 5 for each concentration

We therefore tested whether *D. suzukii* ac3 responses to 1-octen-3-ol, 2-heptanone, hexanal, and *E*-2-hexenal depend on Orco. To address this, we used a *D. suzukii Orco*^*3*^ mutant. This mutant was generated using CRISPR/Cas9 technology [6]. We compared the responses in all four types of coeloconic sensilla between wild-type (+ */* +) and *Orco* mutant (*Orco*^*3*^) flies (Fig. 3).

We found, as expected, that the response profiles of ac1, ac2, and ac4 were comparable between the two genotypes (p > 0.05 for each odorant, Mann–Whitney test) (Fig. 3A–C). However, the response profile of the ac3 sensilla exhibited significant differences, especially the responses to 1-octen-3-ol, 2-heptanone, *E*-2-hexenal, and hexanal. In *D. suzukii Orco*^*3*^ mutant flies, the responses to each of these four odorants were markedly reduced compared to *D. suzukii* wild-type flies (+ / + (*p* < 0.0001 for 1-octen-3-ol, *p* = 0.007 for 2-heptanone, hexanal, and *p* = 0.003 for *E*-2-hexenal; Mann–Whitney test) (Fig. 3A, C). By contrast, the responses to the remaining odorants remained unaffected (*p* > 0.05 for each odorant; Mann–Whitney test) (Fig. 3B, C).

We next screened the ac3 sensilla in both *D. suzukii* wild-type (+ / +) and *Orco*^*3*^ mutant flies with five different concentrations of each of 1-octen-3-ol, 2-heptanone, *E*-2-hexenal, and hexanal. We found that at each concentration where any of these four odorants elicited responses in *D. suzukii* wild-type flies, the responses were diminished in *D. suzukii orco*^*3*^ mutant flies (*p* < 0.05; Mann Whitney test; *n* = 5) (Fig. 3D).

These findings indicate that responses of the ac3 sensilla in *D. suzukii* to 1-octen-3-ol, 2-heptanone, *E*-2-hexenal, and hexanal require Orco, while responses to the remaining odorants in the ac3 sensilla, as well as the responses of the ac1, ac2, and ac4 sensilla to their respective odorants, do not require Orco.

### Differences in electrophysiological responses of coeloconic sensilla between *D. suzukii* and its close relatives

We next tested whether the responses of coeloconic sensilla in *D. suzukii* differ from those of its close relatives. To address this, we included *D. biarmipes* and *D. melanogaster* in our analysis. *D. biarmipes* stands closer in evolutionary relation to *D. suzukii* than to *D. melanogaster* [37]. This species exhibits no preference for either ripe or overripe fruits [4, 6, 38].

**Fig. 4:**
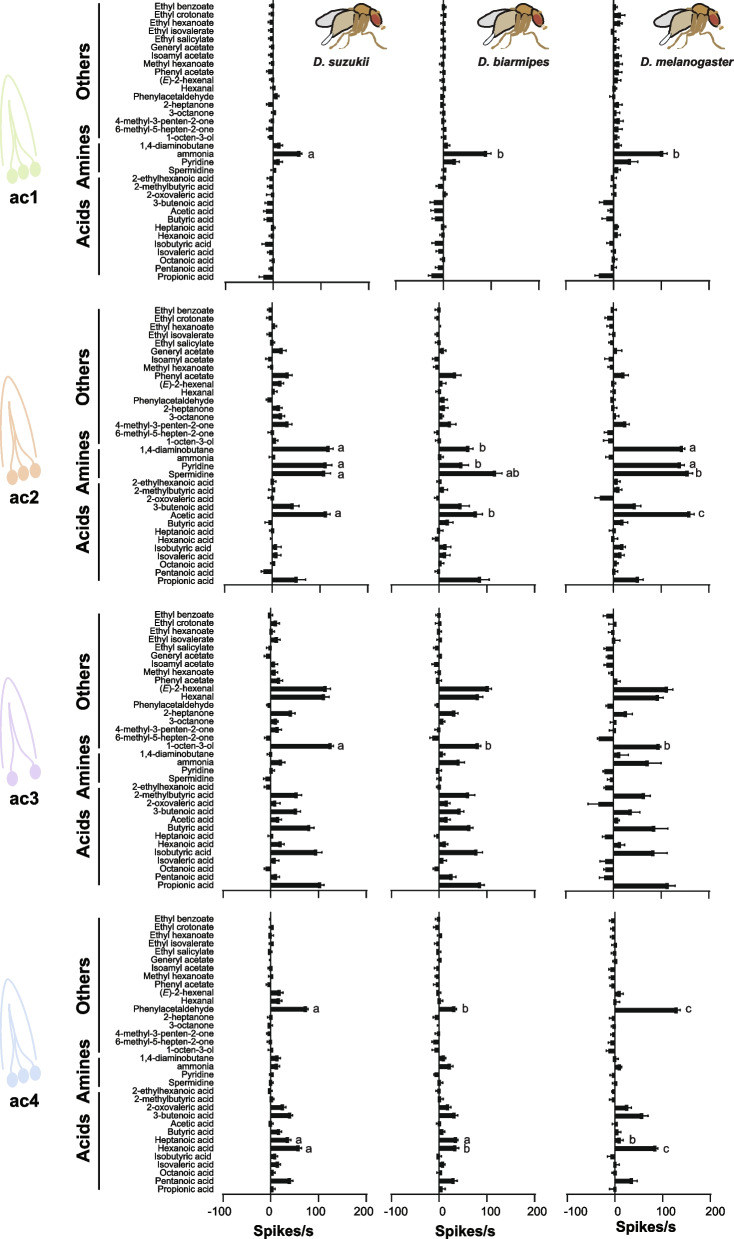
Responses of coeloconic sensilla in *D. suzukii*, *D. biarmipes*, and *D. melanogaster* mated females. Error bars represent means ± SEM. For *D. suzukii* mated females, sample sizes (*n*) range from 12 to 31. For *D. biarmipes* mated females, sample sizes (*n*) range from 5 to 14. For *D. melanogaster* mated females, sample sizes (*n*) range from 5 to 10. Odorants were diluted 10.^−2^ in double-distilled water. Responses to the diluent control, water, were subtracted from each value. One-way ANOVA followed by Tukey’s multiple comparison test. Values indicated with different letters are significantly different (*p* < 0.05)

While potent ligands and many responses remained consistent across all three species, we observed differences in the responses to nine odorants (Fig. 4). In the ac1 sensilla, responses to ammonia were reduced in *D. suzukii* compared to the other two species (adjusted *p* < 0.05; One-way ANOVA followed by Tukey’s multiple comparison test).

**Fig. 5:**
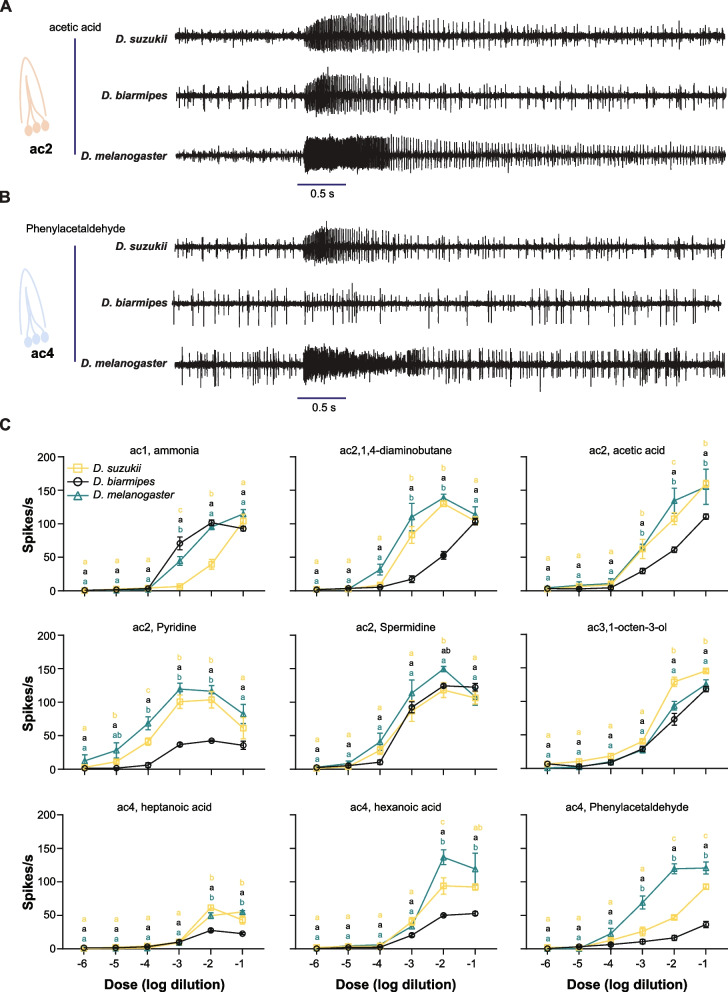
Differences in electrophysiological responses across species are dose dependent. **A** Example traces of electrophysiological responses to acetic acid (10^−2^ dilution) in *D. suzukii*, *D. biarmipes*, and *D. melanogaster* mated females*.* **B** Example traces of electrophysiological responses to phenylacetaldehyde (10^−2^ dilution) in *D. suzukii*, *D. biarmipes*, and *D. melanogaster* mated females*.* **C** Responses to different dilutions of the indicated odorants in *D. suzukii*, *D. biarmipes*, and *D. melanogaster* mated females. Error bars represent means ± SEM. Odorants were diluted 10.^−2^ in double-distilled water. *n* = 5 in each species for each concentration. One-way ANOVA followed by Tukey’s multiple comparison test. Values indicated with different letters are significantly different (*p* < 0.05)

In the ac2 sensilla, responses to 1,4-diaminobutane and pyridine were decreased in *D. biarmipes* compared to *D. suzukii* and *D. melanogaster*, while responses to spermidine were reduced in *D. suzukii* compared to *D. melanogaster* but not to *D. biarmipes* (adjusted *p* < 0.05; One-way ANOVA followed by Tukey’s multiple comparison test). Additionally, in the ac2 sensilla, responses to acetic acid varied among all three species. *D. biarmipes* showed lower responses to acetic acid than *D. melanogaster,* while the responses of *D. suzukii* were intermediate (adjusted *p* < 0.05; One-way ANOVA followed by Tukey’s multiple comparison test). The duration of the responses to acetic acid was also shorter in both *D. suzukii* and *D. biarmipes* than in *D. melanogaster* (Fig. 5A)*.*

In the ac3 sensilla, 1-octen-3-ol produced stronger responses in *D. suzukii* than in *D. melanogaster* or *D. biarmipes* (adjusted *p* < 0.05; one-way ANOVA followed by Tukey’s multiple comparison test).

In the ac4 sensilla, *D. biarmipes* and *D. suzukii* exhibited a severe reduction in responses to phenylacetaldehyde and hexanoic acid compared to *D. melanogaster*, while responses to heptanoic acid increased in both *D. suzukii* and *D. biarmipes* relative to *D. melanogaster* (adjusted *p* < 0.05; one-way ANOVA followed by Tukey’s multiple comparison test).

We next tested whether these differences could also be observed across various concentrations. For each odorant, we tested six dilutions ranging from 10^−6^ to 10^−1^ (Fig. 5A–C). This analysis indicated that these differences were dose dependent. This analysis also indicated that all three species responded differently to at least one concentration of each of the five odorants (adjusted *p* < 0.05; one-way ANOVA followed by Tukey’s multiple comparison test) (Fig. 5C): ammonia-ac1 at 10^−3^ dilution, acetic acid-ac2 at 10^−2^ dilution, pyridine-ac2 at 10^−4^, hexanoic acid-ac4 at 10^−2^ dilution, and phenylacetaldehyde-ac4 at 10^−2^ and 10^−1^ dilutions.

This analysis also identified several species-specific differences. For example, *D. suzukii,* compared to *D. melanogaster* and *D. biarmipes*, exhibited reduced responses to ammonia at 10^−2^ dilution in ac1 and increased responses to 1-octen-3-ol at 10^−1^ and 10^−2^ dilutions in ac3 (adjusted *p* < 0.05, One-way ANOVA followed by Tukey’s multiple comparison test) (Fig. 5C).

These analyses collectively uncovered variations in the peripheral detection of several odorants that act on neurons within coeloconic sensilla between *D. suzukii* and its relatives, *D. biarmipes* and *D. melanogaster*. These analyses also revealed that some of these differences are unique to *D. suzukii*, while others are shared between *D. suzukii* and *D. biarmipes*. These distinctions likely facilitate adaptation to the ecological niche of each species.

We acknowledge that strains other than those used in this study may display responses different from those observed in our study.

### Olfactory preferences of *D. suzukii* and its relatives to odorants that act on coeloconic sensilla

**Fig. 6:**
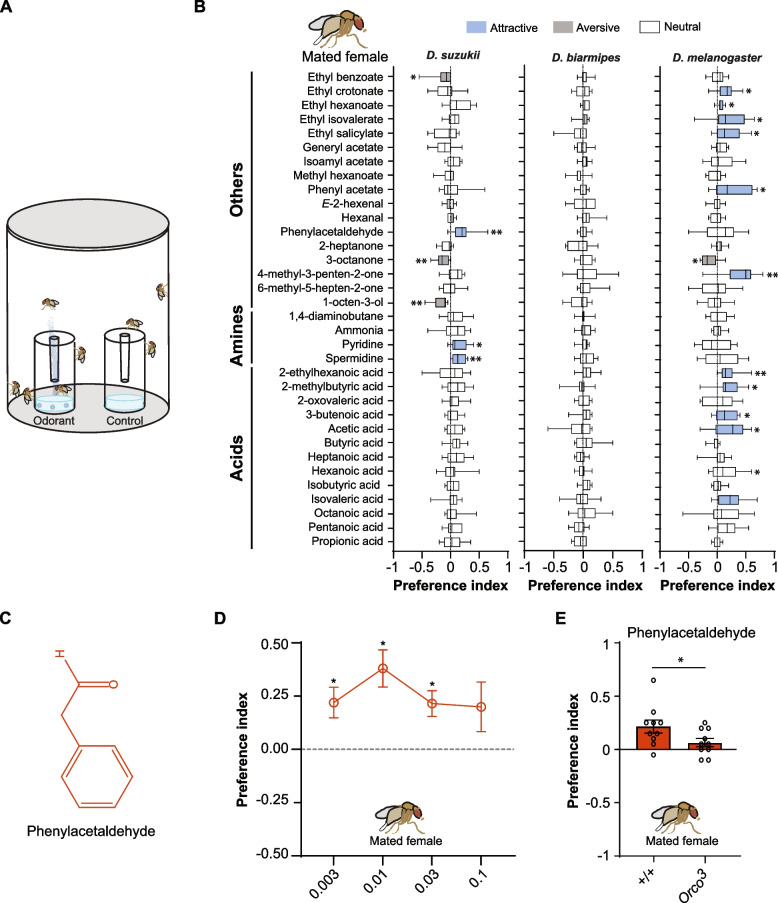
Olfactory attraction of *D. suzukii*,* D. biarmipes*, and *D. melanogaster*. **A** Schematic of the olfactory preference trap assay. **B** Olfactory preference indices of *D. suzukii*, *D. biarmipes*, and *D. melanogaster* mated females to 34 odorants. Boxplots depict median responses, interquartile ranges, and maximum and minimum values. Each odorant was tested at 0.03% concentration. One-sample Wilcoxon test was used to determine whether a response was statistically different from zero. *p < 0.05, **p < 0.01, *n* = 10 for each odorant. Please note that if we corrected the *p*-values using Benjamini–Hochberg correction, none of the responses to the tested odorants in any of the three species would remain statistically significant. However, if we focused our analysis on the nine compounds that showed differential electrophysiological responses across the species, as demonstrated in Fig. 5, the *p*-value for each of 1-octen-3-ol (*p* = 0.02), phenylacetaldehyde (*p* = 0.03), spermidine (*p* = 0.02), and pyridine (*p* = 0.04) would remain statistically significant. **C** Chemical structure of phenylacetaldehyde. **D** Behavioral responses of *D. suzukii* mated females to different percent concentrations of phenylacetaldehyde. Error bars represent means ± SEM. One-sample Wilcoxon test with Benjamini–Hochberg correction was used to determine whether a response was statistically different from zero. *p < 0.05, *n* = 10 for each concentration. **E** Behavioral response of *D. suzukii* wild-type (+ / +) and *Orco*^*3*^ mutant mated females to 0.03% phenylacetaldehyde. Error bars represent means ± SEM. To test whether the two genotypes differ from each other, we used a Mann–Whitney test (*p* ≤ 0.05, *n* = 10 for each genotype)

We next examined the olfactory preferences of *D. suzukii* and its relatives, *D. biarmipes* and *D. melanogaster,* towards our battery of 34 odorants. We aimed to determine if any of these odorants elicit attraction in any of the tested species that is significantly different from zero. For this purpose, we used a two-choice trap assay (Fig. 6A). In this essay, flies were freely moving within a plastic pot that contained two distinct traps. One of the traps contained a mixture of agar, sucrose, and a solvent control, while the other contained the same agar-sucrose base combined with a test odorant. Importantly, in this assay, flies could not access the trap’s contents until they entered it.

We found that *D. suzukii* exhibited attractive responses to three odorants (phenylacetaldehyde, pyridine, and spermidine) and aversive responses to three other odorants (1-octen-3-ol, 3-octanone, and ethyl benzoate) (*p* < 0.05; one-sample Wilcoxon test; *n* = 10 for each odorant) (Fig. 6B). Notably, 1-octen-3-ol was also found to be aversive to *D. suzukii* in both laboratory choice tests and agricultural settings [39]. In our electrophysiological analysis, 1-octen-3-ol elicited stronger responses in ac3 sensilla in *D. suzukii* than in *D. biarmipes* or *D. melanogaster*.

By contrast, *D. melanogaster* was attracted to 11 odorants and avoided one odorant (3-octanone) (*p* < 0.05; one-sample Wilcoxon test; n = 10 for each odorant) (Fig. 6B). The attractive odorants included five esters (ethyl crotonate, ethyl hexanoate, ethyl isovalerate, ethyl salicylate, and phenyl acetate), one ketone (4-methyl-3-penten-2-one), and five acids (2-ethylhexanoic acid, 2-methylbutyric acid, 3-butenoic acid, acetic acid, and isovaleric acid. *D. biarmipes*, on the other hand*,* showed no attraction or aversion to any of the tested odorants (*p* > 0.05; one-sample Wilcoxon test; *n* = 10 for each odorant) (Fig. 6B).

We further confirmed the attraction of *D. suzukii* to phenylacetaldehyde by testing a range of concentrations: 0.003, 0.01, 0.03, and 0.1%. This dose–response experiment revealed that *D. suzukii* mated females were significantly attracted to three concentrations of phenylacetaldehyde (*p* < 0.05; one-sample Wilcoxon test with Benjamini–Hochberg correction; *n* = 10 for each concentration) (Fig. 6C, D).

Altogether, these results demonstrate that *D. suzukii* has evolved a unique attraction to certain odorants, such as phenylacetaldehyde, and no longer shows attraction to odorants that are important to *D. melanogaster*, such as acetic acid [40, 41]. These behavioral differences may represent evolutionary chemosensory adaptations in *D. suzukii* to facilitate its transition to ripe fruits.

### Attraction of *D. suzukii* to phenylacetaldehyde requires Orco

Finally, we aimed to determine whether the attraction to phenylacetaldehyde in *D. suzukii* depends on the Ir-expressing or Or-expressing olfactory system. In *D. melanogaster*, phenylacetaldehyde strongly activates both systems at the cellular level [31, 42]. To investigate this, we used *D. suzukii Orco*^*3*^ mutant. In this mutant, the Ir-expressing olfactory system remains fully functional, while the Or-expressing olfactory system is non-functional.

We found that the response of *Orco*^*3*^ mutant mated females was significantly reduced compared to that of *D. suzukii* wild-type mated females (Mann–Whitney test, *p* < 0.05, *n* = 10) (Fig. 6E). This finding demonstrates that the attraction of *D. suzukii* to phenylacetaldehyde requires the Or-expressing system.

## Discussion

### Coeloconic sensilla in *Drosophila suzukii*

We have functionally characterized the antennal coeloconic sensilla in the global fruit pest *D*. suzukii and found that they fall into four functional types. These types were identified by screening 78 coeloconic sensilla in ~ 20 mated females using the single-sensillum recording technique, a battery of 34 odorants, hierarchical cluster analysis, and principal component analysis followed by One-way ANOSIM. These types respond to distinct odorants and occupy specific locations on the antenna.

We also found that the ac3 sensilla in *D. suzukii* responds strongly to various acids, alcohols, ketones, and aldehydes. These alcohols, ketones, and aldehydes are the primary activators of *D. melanogaster* Or35a, which is expressed in ac3B neurons [20–22].

Across *Drosophila* species, the ac3 sensilla exhibits two distinct response profiles [9, 21, 22, 26]. The first response profile resembles that found in *D. suzukii*. This response profile is also found in *D. melanogaster*, *D. simulans*, *D. sechellia*, *D. yakuba*, *D. erecta*, and *D. biarmipes* (this study) [9, 20–22, 26]. The second response profile lacks responses to aldehydes, alcohols, esters, and ketones, likely due to the absence of Or35a expression in this sensillum type [26], while still showing responses to acids. This response profile is found in *D. ananassae*, *D. persimilis*, *D. arizonae*, and *D. mojavensis* [26]. Our results thus indicate the presence of an ac3 sensillum type in *D. suzukii* that responds to the primary activators of *D. melanogaster* Or35a. This finding is further supported by the detection of an Or35a ortholog in the transcriptomic analyses of *D. suzukii* antennae [32, 36, 43, 44].

### Responses to alcohols, ketones, and aldehydes in *D. suzukii* ac3 sensilla depend on Orco

In *D. melanogaster*, the neurons (ac3B) of the ac3 sensilla that respond to aldehydes, alcohols, esters, and ketones express Or35a along with Orco [20–22]*.* The role of Or35a in the responses to these odorants has been demonstrated in *D. melanogaster* [22]. However, the involvement of Orco in these responses remains largely unexplored in *D. melanogaster*, *D. suzukii*, or any other *Drosophila* species. Vulpe and Menuz [23] have reported the dependence of the response to only one odorant in *D. melanogaster* ac3B neurons on Orco. In our study, we tested a *D. suzukii Orco* mutant line and found that the ac3 sensilla of this mutant line exhibited severely reduced responses to each of 1-octen-3-ol (an alcohol), 2-heptanone (a ketone), *E*-2-hexenal (an aldehyde), and hexanal (an aldehyde) at one or more concentrations, while responses to propionic acid, butyric acid, isobutyric acid, 3-butenoic acid, and 2-methylbutyric acid remain intact. These results suggest that Or35a likely operates with Orco, as expected, to form an olfactory detector for alcohols, aldehydes, and ketones in the ac3 sensilla of *D. suzukii*.

### Changes in sensitivity to acetic acid and phenylacetaldehyde between *D. suzukii* and its close relatives

We also found differences in physiological and behavioral responses to some odorants between *D. suzukii* and its relatives *D. biarmipes* and *D. melanogaster*. Of particular interest is the change in responses to acetic acid. Acetic acid, a key indicator of microbial fermentation, is found in fermented fruits at concentrations around 3.5% [45]. Acetic acid is also found in ripe fruit at concentrations between 0 and 1% [45–47].

Our study revealed that both *D. suzukii* and *D. biarmipes* exhibited reduced responses to acetic acid in the ac2 sensilla when compared to *D. melanogaster*. This reduction in physiological responses is consistent with a decreased attraction to acetic acid in both species*,* relative to *D. melanogaster*. Reduced or no response to acetic acid in the ac2 sensilla has also been reported in *D. sechellia*, which has also lost attraction to acetic acid, as well as in *D. willistoni*, *D. mojavensis*, and *D. virilis* [9, 26, 27]. Interestingly, in these species, the decline in responses to acetic acid has been accompanied by increased responses to other acids, such as butyric acid and propionic acid [9, 26, 27]. However, in *D. suzukii*, no increase in responses to other acids was observed.

The reduced response to acetic acid in the ac2 sensilla may not be the sole change that contributes to the decreased attraction of *D. suzukii* to acetic acid. Changes in taste responses could also play a role. In *D. melanogaster,* high levels of acetic acid activate bitter neurons, triggering avoidance behavior [48–50]. The taste ionotropic receptor Ir7a*,* expressed in these bitter neurons, is tuned to detect acetic acid exclusively and is crucial in mediating this avoidance [50]. Intriguingly, this receptor is under positive selection in *D. suzukii* [38], and alterations in its sensitivity or response magnitude may further contribute to the decreased attraction of *D. suzukii* to acetic acid.

Additionally, we cannot exclude the contribution of Ir64a-expressing neurons in the behavioral differences between *D. suzukii* and *D. melanogaster* in response to acetic acid. These neurons are housed in coeloconic sensilla that are in the third chamber of the sacculus. In *D. melanogaster*, these neurons respond to various acids, including acetic acid, and mediate avoidance behavior towards these acids [51, 52]. However, no studies to date have reported that this gene is under any form of selection in *D. suzukii* [38, 53].

The difference in responses to phenylacetaldehyde is also intriguing. Phenylacetaldehyde is an aromatic compound found in a diverse range of fruits, including the host fruits of *D. suzukii* [42, 54, 55]. Phenylacetaldehyde is used in the fragrance industry to impart a floral scent to perfumes, cosmetics, and household products [42].

Phenylacetaldehyde, in our study, elicited lower responses in the ac4 sensilla of both *D. suzukii* and *D. biarmipes* compared to those of *D. melanogaster*. These reduced responses were consistent across two different dilutions (10^−1^ and 10^−2^). Interestingly, despite the decreased responses in ac4 sensilla, phenylacetaldehyde induced strong attraction in *D. suzukii* mated females across a range of concentrations.

In *D. melanogaster*, phenylacetaldehyde strongly activates two olfactory receptor neurons (ORNs): Ir84a-expressing neurons of the ac4 sensilla, which express the male-specific isoforms of the transcription factor Fruitless, and Or67a-expressing neurons of the antennal basiconic sensillum type 10 (ab10) [21, 22, 31, 42]. In *D. melanogaster*, the activation of both neurons, and possibly others, by phenylacetaldehyde promotes male courtship but not male or female attraction [21, 42]. By contrast, *D. suzukii* exhibited reduced responses in the ac4 sensilla and robust attraction to phenylacetaldehyde.

The robust attraction of *D. suzukii* to phenylacetaldehyde could be due to a change in the odorant receptor Or67a and its neurons, alongside the reduced responses in the ac4 sensilla. This hypothesis is supported by our finding that the attraction to phenylacetaldehyde requires Orco (Fig. 6E). Interestingly, *D. suzukii* has evolved five copies of Or67a due to rapid adaptive protein evolution [1, 56]. This expansion could lead to a higher number of neurons responsive to phenylacetaldehyde or expression of the Or67a copies in different neuron types. One or more of these neurons could mediate the strong attraction to phenylacetaldehyde.

Thus, it is likely that phenylacetaldehyde, along with the changes in its peripheral detection described or suggested in this study, and possibly changes in downstream circuitries, as those discovered in the noni specialist *D. sechellia* [2, 10], have enabled *D. suzukii* to shift its preference for ripe fruits. It will be interesting, in the future, to determine how phenylacetaldehyde is detected and encoded by the *D. suzukii* olfactory receptor neurons that express Or67a copies. Creating CRISPR mutants for these Or67a copies would allow the assessment of the specific roles of these paralogs in mediating attraction to phenylacetaldehyde and any consequent shifts in fruit preference.

### Attractants and repellents for managing *D. suzukii*

We also identified three specific attractants for *D. suzukii*: phenylacetaldehyde, pyridine, and spermidine. Each of these odorants attracted *D. suzukii *mated females but did not attract *D. melanogaster* or *D. biarmipes *mated females, indicating their specificity to *D. suzukii*.

The identification of these specific attractants may significantly advance the development of targeted traps for *D. suzukii*. One of the main issues with current traps and lure technology for this species is their low specificity, which leads to the capture of large numbers of non-target *Drosophila* species [57]. This lack of specificity not only reduces the efficiency of the traps but also increases the time and labor required for *D. suzukii* management, as non-target species must be sorted and discarded. By incorporating phenylacetaldehyde, pyridine, and spermidine into trap designs, it is possible to create more effective and efficient traps that specifically attract *D. suzukii*.

Additionally, the three repellents identified (1-octen-3-ol, 3-octanone, and ethyl benzoate) in this study can be integrated with the attract-kill strategy [58, 59]. This strategy lures the pest to the bait without touching the edible parts of the crop plants. This dual strategy not only enhances the specificity of traps but also employs repellents to push *D. suzukii* away from valuable crops.

By leveraging specific attractants to draw the pests into traps and repellents to keep them away from crops, this method maximizes efficiency and minimizes collateral damage to non-target species. This innovative approach holds great promise for improving the management of *D. suzukii* infestations, ultimately protecting crops more effectively and sustainably.

One limitation of our study is the relatively low statistical power (0.4119), which should be considered when interpreting our findings, as some differences may not have been detected due to insufficient sample sizes. Future studies with larger sample sizes and increased statistical power would be valuable in further elucidating the olfactory adaptations of *D. suzukii*.

## Conclusion

In summary, we found that the shift in preference of the global fruit pest *D. suzukii* from overripe to ripe fruits has been accompanied by changes in sensitivity to key odorants in coeloconic sensilla. These changes include decreased physiological and behavioral responses to certain fermentation products, such as acetic acid, which play a crucial role in *D. melanogaster*'s preference for overripe fruits. Additionally, there is a gain of unique attraction, accompanied by decreased physiological responses, to phenylacetaldehyde, a compound common in fruits and flowers. These alterations, along with other peripheral and central changes, likely contribute to the novel behavior of *D. suzukii*. Our work also identified phenylacetaldehyde as a potent attractant for *D. suzukii*, which can be useful for developing new lures to mitigate its impact on the fruit industry.

## Methods

### *Drosophila* stocks

*D. melanogaster Canton-S*, *D. suzukii*, *D. suzukii Orco*^*3*^ mutant, and *D. biarmipes* were reared on corn syrup and soy flour culture medium (Archon Scientific) at 24 °C and 50% relative humidity in a 12:12-h light–dark cycle. *D. melanogaster* Canton-S stock used in this study was CS-5 described by Monte et al. [60]. *D. suzukii* stock was collected in Connecticut. *D. biarmipes* (14,023–0361.04) stock was obtained from the *Drosophila* Species Stock Center. *D. suzukii Orco*^*3*^ mutant line was obtained from Dr. Benjamin Prud’homme. Flies aged 5–7 days were used in all experiments.

### Odorants

Chemicals of the highest available purity were obtained from Millipore Sigma or TCI America and stored as recommended. The chemicals and their catalog numbers are ethyl benzoate (Cat.# E12907), ethyl crotonate (TCI America, Product # C0418), ethyl hexanoate (Cat.# 148,962), ethyl isovalerate (Cat.# 71,607), ethyl salicylate (Cat.# 68,291), geranyl acetate (Cat.# 173,495), isoamyl acetate (Cat.# W205532), methyl hexanoate (Cat.# 259,942), phenyl acetate (Cat.# 108,723), *E*-2-hexenal (Cat.# 132,659), hexanal (Cat.# 18,109), 2-heptanone (Cat.# 537,683), 3-octanone (Cat.# 136,913), 4-methyl-3-penten-2-one (Cat.# 49,722), 1-octen-3-ol (Cat.# O5284), 1,4-diaminobutane (Cat.# D13208), ammonium hydroxide solution (Cat.# 338,818), pyridine (Cat.# 27,040), spermidine (Cat.# S2626), 2-ethylhexanoic acid (Cat.# 538,701), 2-methylbutyric acid (Cat.# 193,070), 2-oxovaleric acid (Cat.# 75,950), 3-butenoic acid (Cat.# 134,716), acetic acid (Cat.# A6283), butyric acid (Cat.# B103500), heptanoic acid (Cat.# W334804), hexanoic acid (Cat.# 153,745), isobutyric acid (Cat.# W222208), isovaleric acid (Cat.# 129,542), octanoic acid (Cat.# C2875), pentanoic acid (Cat.# 75,054), propionic acid (Cat.# 402,907), and phenylacetaldehyde (Cat.# W287407). For electrophysiology and behavioral experiments, all chemicals were diluted in water.

A volume of ten microliters of each odorant was pipetted onto a 1.3 cm diameter disc of filter paper, which was placed into the large end of a disposable borosilicate glass Pasteur pipette (2 ml volume). This Pasteur pipette was then inserted, with the tip (the narrow end) of the pipette passing through a hole in a glass tube carrying a humidified air stream (1 l/min) directed at the fly. A 0.5-s pulse of air (500 ml/min) was administered through the pipette containing the odorant. Odorants were presented one after the other with an interval of at least 60 s between the delivery of each odorant. For dose–response curves, odorants were presented with increasing doses in log steps.

### Electrophysiology

A single fly was placed in a 200-μL plastic pipette tip with its head directed towards the narrower end to allow only the antennae to protrude. The pipette tip was then securely attached to a glass microscope slide. The antenna was gently stabilized on a cover slip using a glass capillary. The slide was then placed under a light microscope (BX51WI, Olympus, Tokyo, Japan) equipped with a 50 × objective (LMPLFLN 50X, Olympus) and 10 × eyepieces. A reference tungsten electrode (catalog no. 716000, A-M Systems), electrolytically sharpened to 1 μm tip diameter by dipping it repeatedly in a 10% KNO3 solution, was inserted into the eye. The recording tungsten electrode, identical to the reference electrode, was inserted gently into the base of a coeloconic sensillum. Signals were amplified (10 × ; Syntech Universal AC/DC Probe; http://www.syntech.nl), sampled (10,667 samples s^−1^), and filtered (100–3000 Hz with 50/60 Hz suppression) via a USB-IDAC connection (Syntech) to a computer. Action potentials were extracted using Syntech AutoSpike 32 software. Responses as the increase (or decrease) in the action potential frequency (spikes/s) were calculated by subtracting the number of action potentials during the 0.5 s preceding the odor stimulation from the number of action potentials during the 0.5 s of odor stimulation.

Initially, we recorded 78 coeloconic sensilla, including 15 ac1 sensilla, 12 ac2 sensilla, 20 ac3 sensilla, and 31 ac4 sensilla, from approximately 20 mated females. This was used to assess variance and determine the adequate sample size. Accordingly, in the other experiments, we generally aimed to have 5–15 replicates. The Wilcoxon test, Mann–Whitney test, and one-way ANOVA followed by Tukey’s multiple comparison test used in this study account for these differences in sample sizes [61].

### Two-choice trap assay

The two-choice trap assay consisted of a plastic pot with a snap lid (https://shop.bugdorm.com/, 960 ml, nylon screen) containing two trap cups (ThermoFisher Scientific, Cat.# 060181). The traps are made from virgin polypropylene vials and white screw caps. The vials (20 ml capacity) measure 4.3 cm in height and have a diameter of 3.5 cm. The screw caps are 1.1 cm in height with a diameter of 3.5 cm. Each cap has a 0.5-cm diameter hole through which a 1-mL filter tip entry extends into the trap. One cup contained 1% agar (Fisher, Cat.# DF0140-01–0) mixed with 2% sucrose (Millipore Sigma, Cat.# S7903), and the second cup contained 1% agar mixed with 2% sucrose and a test odorant (Fig. 6A). Twenty fed, mated female flies were introduced into the plastic pot, which was then closed with the lid and left for 24 h in the dark. Flies enter traps via a 1-mL filter tip that is inserted through a hole in the middle of the trap cap. A preference index was calculated as (number of flies in the trap containing 1% agar mixed with 2% sucrose and a test odorant − number of flies in the trap containing 1% agar mixed with 2% sucrose)/(total number of flies). From the beginning, we aimed for 10 replicates for each odorant and concentration in every species. We acknowledge that the statistical power for this number of replicates is 0.4119, as determined by a post hoc power analysis.

### Experimental design and statistical analysis

Hierarchical cluster analysis using Ward’s method [62] and principal component analysis [63] were performed with PAST (paleontological statistics software package for education and data analysis) [64]. These techniques organize the data into clusters based on the response profiles of each sensillum to the panel of odorants. The Heatmap was also generated in PAST. We also used PAST to calculate the One-way ANOSIM (ANalysis Of Similarities), which is a non-parametric test of significant difference between two or more groups, based on any distance measure [65]. Other statistical tests were performed in GraphPad Prism (version 10.0.1 (316)).

## Data Availability

Data is provided within the manuscript.

## Abbreviations

SWD: Spotted Wing Drosophila
Orco: Odorant receptor co-receptor
Ors: Odorant receptors
Irs: Ionotropic receptors
ac1: antennal coeloconic type 1
ac2: antennal coeloconic type 2
ac3: antennal coeloconic type 3
SSR: Single-eSnsillum Recording
ANOSIM: ANalysis Of Similarities
PCA: Principal Component Analysis
